# Analysis of brain and spinal MRI measures in a common domain to investigate directional neurodegeneration in motor neuron disease

**DOI:** 10.1007/s00415-022-11520-1

**Published:** 2022-12-12

**Authors:** C. Toh, A. Keslake, T. Payne, A. Onwuegbuzie, J. Harding, K. Baster, N. Hoggard, P. J. Shaw, I. D. Wilkinson, T. M. Jenkins

**Affiliations:** 1 Sheffield Institute for Translational Neuroscience, University of Sheffield, Sheffield, UK; 2 School of Mathematics and Statistics, University of Sheffield, Sheffield, UK; 3 Academic Unit of Radiology, University of Sheffield, Sheffield, UK; 4 Sheffield Teaching Hospitals NHS Foundation Trust, Sheffield, UK; 5 Royal Perth Hospital, Victoria Square, Perth, WA 6000 Australia

**Keywords:** Motor neuron disease, Amyotrophic lateral sclerosis, MRI, Neurodegenerative disease

## Abstract

**Background:**

Magnetic resonance imaging (MRI) of the brain and cervical spinal cord is often performed in diagnostic evaluation of suspected motor neuron disease/amyotrophic lateral sclerosis (MND/ALS). Analysis of MRI-derived tissue damage metrics in a common domain facilitates group-level inferences on pathophysiology. This approach was applied to address competing hypotheses of directionality of neurodegeneration, whether anterograde, cranio-caudal dying-forward from precentral gyrus or retrograde, dying-back.

**Methods:**

In this cross-sectional study, MRI was performed on 75 MND patients and 13 healthy controls. Precentral gyral thickness was estimated from volumetric T1-weighted images using FreeSurfer, corticospinal tract fractional anisotropy (FA) from diffusion tensor imaging using FSL, and cross-sectional cervical cord area between C1-C8 levels using Spinal Cord Toolbox. To analyse these multimodal data within a common domain, individual parameter estimates representing tissue damage at each corticospinal tract level were first converted to *z*-scores, referenced to healthy control norms. Mixed-effects linear regression models were then fitted to these *z*-scores, with gradients hypothesised to represent directionality of neurodegeneration.

**Results:**

At group-level, *z*-scores did not differ significantly between precentral gyral and intracranial corticospinal tract tissue damage estimates (regression coefficient − 0.24, [95% CI − 0.62, 0.14], *p* = 0.222), but step-changes were evident between intracranial corticospinal tract and C1 (1.14, [95% CI 0.74, 1.53], *p* < 0.001), and between C5 and C6 cord levels (0.98, [95% CI 0.58, 1.38], *p* < 0.001).

**Discussion:**

Analysis of brain and cervical spinal MRI data in a common domain enabled investigation of pathophysiological hypotheses in vivo. A cranio-caudal step-change in MND patients was observed, and requires further investigation in larger cohorts.

## Introduction

Multimodal magnetic resonance imaging (MRI) enables concurrent assessment of several regions of the central nervous system, each technique providing a differing anatomical and pathophysiological perspective. Combination of derived tissue damage parameters enables investigation of mechanistic damage along neural pathways, such as the corticospinal tract. An integrated analysis approach to concatenate heterogeneous measures into a common domain may facilitate hypothesis-driven research, for example, to assess directionality of neurodegeneration in motor neuron disease/amyotrophic lateral sclerosis (MND/ALS) [1]. In this study, we introduce an approach which allows damage metrics derived from volumetric and diffusion brain and cervical spinal MRI to be integrated into a single model that addresses competing hypotheses. Top-down, dying-forward [2–9] neurodegeneration starting within the precentral gyrus and bottom-up, dying-back [10–16] from the anterior horn cell have each been proposed from previous experimental, pathological, neurophysiological and radiological studies. We designed an approach to enable this longitudinal process to be investigated using a cross-sectional dataset, avoiding attrition bias towards slow progressors.

MRI is often performed routinely at diagnosis of MND/ALS primarily to exclude mimics, but also provides established group-level markers of tissue damage. Primary motor cortical thinning on T1-weighted MRI [17–24], decreased fractional anisotropy (FA) within corticospinal tracts on diffusion tensor imaging [14, 25–35] and reduced cross-sectional area of the cervical spinal cord [36–40] have been consistently reported. The aim of this study was to determine whether analysis of these metrics in a common domain could demonstrate group-level gradients of tissue damage to support either a dying-forward or dying-back hypothesis. To achieve this objective, imaging measures of tissue damage were first converted to dimensionless z-scores (a common domain for analysis), then entered into linear regression models, specifying cranio-caudal location along the corticospinal tract. The hypothesis was that a significant positive gradient would indicate top-down dying-forward neurodegeneration, whilst a negative gradient would indicate dying-back.

## Methods

Ethical approval was obtained (South Yorkshire and the Humber REC reference 13/YH/027 (2/10/2013)) and written informed consent was obtained from all participants.

### Participants

Seventy-five consecutive patients with MND were recruited at time of diagnosis from the tertiary referral neuromuscular clinic at the Royal Hallamshire Hospital, Sheffield, UK between 2013 and 2018. Inclusion criteria for patients were a clinical diagnosis of MND made by a consultant neurologist, based on revised El Escorial criteria and after exclusion of mimics [41]. Thirteen healthy controls were recruited by advertisement. Exclusion criteria for both MND patients and healthy controls were contraindications to MRI, a neurological disorder with potential to confound results, or inability to give informed consent. None of the patients had clinically overt frontotemporal dementia; systematic neuropsychological testing was not performed. MRI scans were reviewed by a consultant neuroradiologist to exclude significant confounding pathology. Minor incidental cranial T2 hyperintensities within limits for age and/or minor cervical spondylotic disease considered of no clinico-radiological significance were not considered exclusion criteria. Demographic and clinical data were obtained including anatomical site and time from symptom onset, and neurological examination findings. Genetic testing was performed on a clinical by-case basis, rather than systematically in all patients. Disability was assessed using the patient-reported ALSFRS-R questionnaire [42].

### MRI acquisition

All imaging was performed at 3 Tesla (Philips Ingenia, Best, Netherlands). Acquisitions included: whole-brain T1-weighted imaging (TR = 8.2 ms, TE = 3.8 ms, 340 slices, voxel-size = 1 × 1.2 × 1 mm); whole-brain diffusion tensor imaging (TR = 3100 ms, TE = 96 m, voxel size = 2.5 × 2.5 × 2.5 mm, b0, b800 s/mm^2^); and T2-weighted cervical cord sequences (TR = 2500 ms, TE = 100 ms, voxel size 0.55 × 0.79 × 4 mm, flip angle = 90°, field of view = 180 mm). The cord imaging sequence was a three-dimensional turbo spin-echo with an echo train length of 45, using a Philips dStream head-neck coil without cardiac or respiratory gating. Sense was used with an acceleration factor of 1.2 in both the phase and slice directions, manufacturer default shim settings, saturation bands applied antero-posteriorly in the phase direction and one coronal 40 mm anterior saturation band used to reduce artefact from swallowing. The field of view comprised axial slices prescribed to cover C1/2 superiorly and extending inferiorly 180 mm to cover the cervical spinal cord.

### Motor tract damage parameters

#### Pre-central gyrus cortical thickness

Surface-based cortical thickness measurements were derived using FreeSurfer software (V.5.3.0, http://surfer.nmr.mgh.harvard.edu/) from T1-weighted images [43]. Motion correction and averaging, skull-stripping, automated Talairach transformation, segmentation, intensity normalisation, tessellation, automated topology correction, surface deformation and cortical thickness estimation were performed using the standard processing pipeline [44]. Outputs were visually reviewed for any errors (such as skull-strip failure, pial misplacement) and corrected by re-running the skull-strip using the graph cut segmentation method or adjusting watershed parameters, following standardised published procedures (https://surfer.nmr.mgh.harvard.edu/fswiki/FsTutorial/SkullStripFix_freeview). Seven MND datasets and one control dataset were excluded from the study due to errors in automated pial contouring that could not be corrected using this methodology. Two MND and one control dataset had no MRI brain data available. No manual editing was performed. Left, right and mean precentral gyral cortical thickness estimates were extracted.

#### Corticospinal tract diffusion tensor imaging

The FMRIB Software Library (FSL: http://fsl.fmrib.ox.ac.uk/fsl/fslwiki/) was used for analysis of diffusion tensor data. Eddy current and movement correction and brain extraction were performed using the FSL processing pipeline. Pre-processed data were fitted with a diffusion tensor model using DTIFIT. Tract-based spatial statistics were used to align the data into a common space using the nonlinear registration tool FNIRT, which applies a b-spline representation of the registration warp field resulting in a group-level mean FA skeleton. Each of subject FA data was then projected and transformed to fit the skeleton in MNI152 standard space. Binary masks of the left and right corticospinal tracts were chosen as the regions of interest and created using the John Hopkins University white-matter tractography atlas [45]. Voxel inclusion probability threshold was set at 10%. The corticospinal tract masks were applied to the final FA maps. This resulted in FA values for the left and right corticospinal tracts; means were reported. Visual checks were made to exclude anatomical positioning errors. Two patients had no available cranial FA data.

#### Cervical spinal cord cross-sectional area

Cervical cord analysis was performed using Spinal Cord Toolbox v3.0.3. Motion correction, image segmentation, registration to an atlas-based template and automated analysis of cross-sectional area were performed according to standard processing pipelines (https://sourceforge.net/p/spinalcordtoolbox/wiki/tools/). Each participant’s images were segmented and registered to the PAM50 template spinal cord image generated from 50 healthy controls. Automated segmentation was performed using Propseg [46]. Based on the cord segmentation, the cord was translated and rotated about the centre of segmentation, the BSplineSyn algorithm applied to perform section-wise regularised rigid registration, and vertebral levels identified. Warping parameters were then used to translate back into native space. Cross-sectional area of the spinal cord was corrected for curvature of the spine using the angle of the centreline, the circumference of the segmentation across slices was computed and estimates normalised, and derived from cervical cord levels C1–C8 [38]. Cross-sectional area was not calculable for a single patient at C1 level. At C7 and C8, the algorithm was unable to produce estimates due to limited field of view in 21 and 33 patients, respectively.

### Statistical analysis

To convert each measurement derived from each MRI technique in each anatomical region into a common domain, normality of data was first confirmed using Shapiro-Wilks tests. *Z*-scores were then calculated at individual patient level, at each level of the corticospinal tract (i.e. precentral gyrus thickness, intracranial corticospinal tract FA, and cross-sectional area of each spinal cord level C1 to C8), as estimates of the degree of damage within that anatomical region, using the healthy control group mean and standard deviation at the corresponding level as normative standards*.* As *z*-scores are dimensionless, it was then possible to fit individual trajectories of damage in a common domain, by entering the *z*-scores into a linear regression model as the dependent variable. The independent variables specified in the model were subject (i.e. identification of each individual participant, to model random effects) and location in the corticospinal tract as an ordinal variable (1 = precentral gyrus; 2 = intracranial corticospinal tract; 3 = C1; 4 = C2; 5 = C3; 6 = C4; 7 = C5; 8 = C6; 9 = C7; 10 = C8 cervical cord levels). For example, a *z*-score of − 3 for precentral gyrus cortical thickness, − 2 for intracranial corticospinal tract FA, and − 1 at cervical cord levels would be considered a positive (dying-forward) motor tract damage gradient, and the reverse pattern a negative (dying-back) gradient. The primary outcome of the study was the mean group-level gradient of tissue damage along the corticospinal tract in MND patients. Secondary outcomes were differences in cortical thickness, intracranial corticospinal tract FA and cervical spinal cord cross-sectional area between MND patients and healthy controls.

Between-group comparisons were conducted using SPSS Statistics version 24.0 (IBM, Armonk, NY) and mixed effects regression modelling performed using Stata version 13.1 (StataCorp, TX), specifying unstructured covariance. Independent two-tailed *t* tests were used to assess between-group differences in continuous variables, and Pearson’s Chi-square test was applied for categorical variables.

All results for each analysis were corrected for multiple comparisons using the Benjamini–Hochberg test [47]. Unadjusted p-values together with statistical significance following correction were reported.

## Results

### Participants

Table 1 summarises the demographic and clinical features of the participants, who were assessed at a relatively early stage of disease, with moderate disability. Eight patients had a family history of possible MND/ALS in a first or second degree relative, and a further six patients had a family history of dementia. One of these patients had a C9orf72 and one patient a SOD1 mutation.

**Table 1:** Participant demographic and clinical data

	MND patients (*n* = 75)	Healthy controls (*n* = 13)	*p* value [95% CI]
	Mean ± SD	Mean ± SD	
Gender			
Male	44	7	*p* = 0.745
Female	31	6	
Age (years)	60 ± 13	57 ± 19	*p* = 0.184 [− 14.43, 2.82]
Time between date of first scan and symptom onset (months)	17 ± 17		
Total ALSFRS-R	38 ± 6		
Site of first symptoms			
Arm	47%		
Leg	21%		
Bulbar	23%		
Multi-regional	9%		
Clinical signs at presentation			
Mixed UMN/LMN	92%		
LMN only	5%		
UMN only	3%		

*ALSFRS-R* amyotrophic lateral sclerosis functional rating scale (revised), *CI* confidence interval, *LMN* lower motor neuron, *MND* motor neuron disease, *SD* standard deviation, *UMN* upper motor neuron

### Motor tract damage measures

MND patients had lower mean precentral gyral thickness compared to healthy controls, but this result did not reach statistical significance. Intracranial corticospinal FA was lower in MND patients than in healthy controls, retaining significance after multiple comparisons correction on the left side. There were no significant differences in cervical spinal cord cross-sectional area. Results are reported in Table 2.

**Table 2:** MRI-derived tissue damage metrics in MND patients compared to controls

	Participant status	*N*	Mean ± SD	*p* value
Precentral gyrus thickness (mm)				
Right	MND patients	66	2.35 ± 0.18	0.088
	Controls	11	2.45 ± 0.11	
Left	MND patients	66	2.36 ± 0.15	0.091
	Controls	11	2.45 ± 0.13	
Mean	MND patients	66	2.36 ± 0.16	0.077
	Controls	11	2.45 ± 0.11	
Corticospinal tract FA				
Right	MND patients	73	0.42 ± 0.03	0.022
	Controls	13	0.44 ± 0.02	
Left	MND patients	73	0.42 ± 0.03	0.002*
	Controls	13	0.44 ± 0.02	
Mean	MND patients	73	0.42 ± 0.03	0.007
	Controls	13	0.44 ± 0.02	
Mean cross-sectional cervical cord area (mm^2^)				
Mean C1–C8	MND patients	63	67.00 ± 10.82	0.494
	Controls	13	69.25 ± 10.52	
C1	MND patients	63	71.05 ± 12.30	0.888
	Controls	13	71.78 ± 17.72	
C2	MND patients	64	72.55 ± 15.43	0.796
	Controls	13	74.07 ± 19.51	
C3	MND patients	64	73.11 ± 17.00	0.715
	Controls	13	74.94 ± 16.03	
C4	MND patients	64	67.17 ± 15.81	0.116
	Controls	13	72.68 ± 9.96	
C5	MND patients	60	61.14 ± 15.71	0.165
	Controls	13	66.80 ± 12.19	
C6	MND patients	60	54.81 ± 12.09	0.456
	Controls	13	50.48 ± 19.60	
C7	MND patients	43	50.15 ± 12.32	0.159
	Controls	13	43.25 ± 15.47	
C8	MND patients	31	46.91 ± 12.18	0.480
	Controls	13	43.38 ± 15.78	

*p* values are reported unadjusted for multiple comparisons

Asterisked results retained significance following Benjamini–Hochberg correction

*C* cervical spinal cord level, *FA* fractional anisotropy, *mm* millimetres, *MND* motor neuron disease, *SD* standard deviation

### Modelling directionality of neurodegeneration

**Fig. 1:**
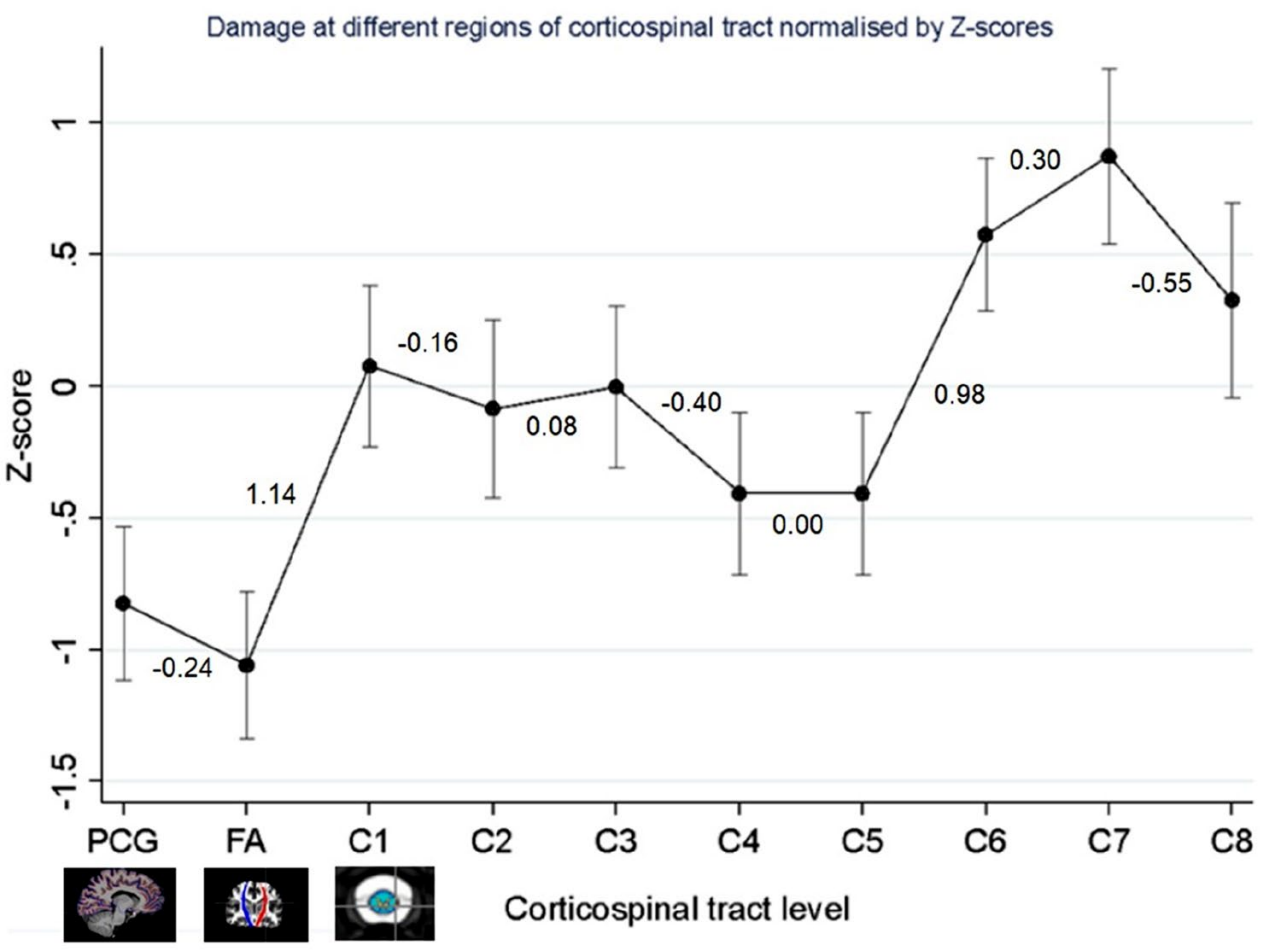
Modelling directionality of neurodegeneration: group-level step-changes between adjacent levels of the corticospinal tract are plotted, derived from mixed effects linear regression. Regression coefficients for the gradient of the slope between each adjacent level of the motor tracts are reported. Error bars represent standard errors. Negative *z*-scores indicate greater tissue damage. *C* Cervical spinal cord level, *PCG* Precentral gyrus cortical thickness, *FA* Fractional anisotropy of the intracranial corticospinal tract

Results are reported in Table 3. There was no significant difference in z-scores between precentral gyral and intracranial corticospinal tract levels (− 0.24, [95% CI − 0.62, 0.14], *p* = 0.222), but a group-level step-change occurred between the intracranial corticospinal tract and C1 level (1.14, [95% CI 0.74, 1.53], *p* < 0.001), with a positive cranio-caudal gradient. A second significant positive cranio-caudal step-change occurred between C5 and C6 levels (0.98, [95% CI 0.58, 1.38], *p* < 0.001) (Fig. 1).

**Table 3:** Modelling directionality of neurodegeneration

Corticospinal tract level	Regression coefficient	95% Confidence interval		*p* value
PCG to intracranial CST	− 0.24	− 0.62	0.14	0.222
Intracranial CST to C1	1.14	0.74	1.53	< 0.001*
C1 to C2	− 0.16	− 0.60	0.27	0.462
C2 to C3	0.08	− 0.35	0.51	0.720
C3 to C4	− 0.40	− 0.81	0.00	0.053
C4 to C5	0.00	− 0.41	0.41	1.000
C5 to C6	0.98	0.58	1.38	< 0.001*
C6 to C7	0.30	− 0.12	0.72	0.165
C7 to C8	− 0.55	− 1.03	− 0.07	0.025

Regression coefficients for the gradients between adjacent anatomical levels of the corticospinal tract derived from mixed effects linear regression are reported, along with the associated 95% confidence interval and *p* value

An asterisk indicates retained statistical significance following Benjamini–Hochberg correction for multiple comparisons

*C* Cervical spinal cord level, *CST* corticospinal tract, *PCG* precentral gyrus

## Discussion

Analysis of volumetric, diffusion, brain and cervical spinal MRI in a common domain identified step-changes in tissue damage measures between the cranial descending corticospinal tract and C1 spinal cord level, and between C5 and C6 cord levels, with an apparent cranio-caudal gradient, but some caution in interpretation is necessary.

The MRI-derived tissue damage metrics from each region are in the range of previously published data for precentral gyral cortical thickness [17, 22–24], intracranial corticospinal tract FA [26, 30, 32, 33] and cervical cord cross-sectional area [36, 37, 39, 40]. A few previous studies have assessed both the brain and cervical spinal cord concurrently in MND/ALS [36, 48–50] and compared changes at different levels, with varying results. A cross-sectional study showed both lower cervical cord cross-sectional area and lower intracranial corticospinal tract FA in MND/ALS patients compared to healthy controls [36]. A longitudinal study found cervical cord cross-sectional area decreased in patients over 9 months, but brain measures did not change [49], whilst another reported that brainstem volume, but not precentral gyral thickness, decreased over 8 months; cord cross-sectional area also decreased and appeared clinically relevant [48]. Differing patterns in sporadic and SOD1 MND/ALS patients have also been reported, with intracranial corticospinal tract FA reduction and cervical cord atrophy predominating, respectively [50]. Longitudinal studies focussed on either brain or cervical cord in isolation have shown progression of intracranial grey [20] and white matter tissue damage [34, 51–53] in some, but not all [17, 54], studies, with only modest cervical cord atrophy evident even in quite large cohorts (mean 0.8 mm^2^ over 6 months in 103 patients, not reaching statistical significance) [40]. Results of studies of asymptomatic gene carriers, which represent an ideal model of the earliest stages of MND/ALS, have reported early detectable changes both at cortical [8] and cord levels [50, 55]. Damage patterns appear to differ between manifest genetic variants [56].

Similarly, cross-sectional studies have reported greater reductions in distal compared to proximal cervical cord [13] and intracranial corticospinal tracts [14, 57], and also the reverse pattern within the brain [26, 30] and spinal cord [58]. An interesting study applied an event-based probabilistic model to characterise evolution of MND/ALS using intracranial corticospinal tract FA, and supported a dying-back process, with earlier distal changes [16]. It is notable that this study investigated longitudinal processes from a cross-sectional dataset, like the present study, but assessed a multi-centre cohort with a much larger control group, focused exclusively on brain motor regions and data required dichotomisation into healthy or diseased states.

The differing results of previous studies may reflect underlying heterogeneity in the pathological process of MND/ALS. Our study adds to the literature by enabling brain and spinal cord measures to be modelled together, rather than reported separately and compared. The observed step-changes could suggest brain pathology predominating in our cohort, or instead represent differences in sensitivity or specificity to detect MND/ALS pathology between the various MRI techniques, an important issue common to all multimodal imaging studies. For example, cross-sectional cervical cord area lacks tract specificity compared to intracranial corticospinal FA. However, this interpretation is not supported by results of previous studies that have applied both techniques [36, 48, 49]. Furthermore, this explanation could not explain the second step-change between C5 and C6, although here estimates may have been biased by smaller participant numbers at the limits of the field of view. Dying-forward neurodegeneration would have been further supported if between-group differences in precentral gyral thickness had been demonstrable, which did not quite reach statistical significance in this cohort. This may reflect type II error due to the small control group, a limitation of our study. Precentral gyral thinning in MND/ALS patients compared to controls has been reported fairly consistently in previous studies [17–23], although again with inter-individual heterogeneity; only half of cases exhibited thinning in one study without detectable group-level differences from healthy controls [59]. In another study, significant differences were demonstrable in some, but not all, clinical sub-phenotypes [60].

Our early cohort included patients with restricted MND phenotypes at the time of assessment, who may have had progressive muscular atrophy or primary lateral sclerosis, with potential to confound results. However, when we repeated the analysis omitting patients with restricted lower motor neuron or upper motor neuron signs, results were materially unchanged (data not shown). No clear pattern of dissociation between upper and lower motor neuron restricted patients was evident on plotting individual regressions (data not shown). Future studies could explore this issue further with larger patient numbers. Analysis could be optimised in future work by parcellating the intracranial corticospinal tract, including more caudal regions of the spinal cord, sub-segmenting the spinal cord by tract to further optimise analysis [51, 54, 61], and collating systematic neuropsychological data. Although the applied cross-sectional approach circumvents the issue of cohort attrition which may bias follow-up observations towards slower progressors, any inferences made on longitudinal processes from a single time-point are necessarily indirect. Longitudinal methodologies are superior to cross-sectional approaches to investigate pathological changes over time [17, 20, 23, 34, 40, 48, 49, 51–53, 55, 58]. Strengths of the current study are the comprehensive coverage of the corticospinal tract using routinely available clinical techniques from a consecutively recruited and relatively large patient cohort, and the combination of brain and cord analysis within a single model.

In summary, analysis of brain and cervical spinal MRI data in a common domain enabled investigation of mechanistic pathophysiological hypotheses in vivo in MND patients. Further work on relative sensitivity of MRI techniques to detect tissue damage using larger healthy control comparator groups may help further refine this modelling approach.
